# Kin selection spreads

**DOI:** 10.7554/eLife.84142

**Published:** 2022-11-24

**Authors:** James P Higham

**Affiliations:** 1 Department of Anthropology, New York University New York United States

**Keywords:** evolution, kin selection, indirect fitness, primate, mandrillus sphinx, Other

## Abstract

By spending more time around infants which physically resemble their own, mandrill mothers may increase how frequently their offspring interact with their paternal half siblings.

**Related research article** Charpentier MJE, Poirotte C, Roura-Torres B, Amblard-Rambert P, Willaume E, Kappeler PM, Rousset F, Renoult JP. 2022. Mandrill mothers associate with infants who look like their own offspring using phenotype matching. *eLife* **11**:e79417. doi: 10.7554/eLife.79417.

Charles Darwin considered the evolution of altruism to be a puzzle (Darwin, 1871). Altruistic acts benefit the recipient but may cost the actor, making it difficult to grasp how they could have evolved in a world where organisms compete to survive. Since individuals who act selfishly should fare better than those with altruistic tendencies, shouldn’t altruism be quickly selected against?

An elegant solution to this problem emerged in the 1930s, when mathematical arguments were laid out to show how genes that enable altruistic behaviors could spread through a population (Fisher, 1930; Haldane, 1932). These ideas were further developed in the 1960s by W.D. ‘Bill’ Hamilton, and later re-derived by George Price (Hamilton, 1964a; Hamilton, 1964b; Price, 1972).

This work showed that if the individual(s) receiving altruistic acts are genetically related to the altruistic actor, and if an altruistic act is sufficiently beneficial to the receivers relative to the costs to the actor, then altruistic behaviors can spread. This process was named ‘kin selection’ (Smith, 1964). A swathe of additional terms were then coined, including ‘indirect fitness’ (the benefits associated with promoting the offspring of relatives) and ‘inclusive fitness’ (the sum total of direct and indirect fitness). These theoretical developments set the stage for thousands of empirical studies of social behavior. Now, in eLife, Marie Charpentier and colleagues in France, Germany and Gabon – including joint first author Clémence Poirotte – report intriguing new evidence which deepens our understanding of how kin selection may take place within complex societies (Charpentier et al., 2022).

The study took place in a free-ranging population of mandrills living in Gabon. These large and colorful ground-dwelling monkeys live in societies structured around groups of closely related females who share a maternal ancestor, with mature males joining the group during the mating season to sire offspring. As for other social mammals, the health and success of an individual is often shaped by their family connections and how well they are integrated in to the community.

Building on their previous work, Charpentier et al. used machine learning to train a human face recognition algorithm on a large database of mandrill face pictures. The program was then used on the study population to show that infants with the same father look alike – more so than unrelated individuals or maternal half-siblings. Long-term behavioral observations then revealed that mothers spend more time close to infants whose faces resemble their own offspring’s. Such patterns may result in paternal half-siblings interacting with each other more frequently, and potentially may lead to the establishment of social relationships between paternally related infants. Lastly, Charpentier et al. build a theoretical model which demonstrates that mothers would gain fitness benefits by promoting altruistic acts among offspring sired by the same father.

Several factors could explain how increased associations may emerge between siblings who share a father. For example, these related infants may have more similar nutritional needs or digestive processes, and hence find themselves in the same feeding areas more often. Charpentier et al. address one such alternative hypothesis: that fathers may be driving these interactions, for example by spending more time with the mothers they have mated with, or with the babies they have sired. They show that this mechanism is unlikely to explain their results, in part because paternal care is thought to be rare or absent, and because many males are not present within the group outside of the mating season.

As the movement of mandrill infants is primarily determined by their mother, Charpentier et al. reason that it is most likely to be maternal behavior that drives interactions between similar-looking infants. While classic kin selection posits that a mother might increase her fitness by interacting with genetically related individuals, the team argues that female mandrills may ensure that their offspring has better survival outcomes by fostering relationships with non-kin individuals (their infant’s paternal half siblings). They term this process ‘second-order kin selection’.

The work by Charpentier et al. features a particularly rich and diverse set of methods, combining digital images, genetics, machine learning, long-term behavioral observations, and theoretical modeling. Together, these approaches result in an impressive analysis and argument which provoke new questions on how indirect fitness effects might manifest, and the kinds of social behaviors that might be explained by kin selection. Overall, this study adds an intriguing new layer to the mechanisms by which kin selection might operate, and to our understanding of the complexity of evolved social behaviors.
